# Effect of *in ovo* supplementation of nano forms of zinc, copper, and selenium on post-hatch performance of broiler chicken

**DOI:** 10.14202/vetworld.2016.287-294

**Published:** 2016-03-19

**Authors:** P. Patric Joshua, C. Valli, V. Balakrishnan

**Affiliations:** 1 Department of Pharmacology, Sri Muthukumaran Medical College Hospital and Research Institute, Dr. M.G.R. Medical University, Chennai, Tamil Nadu, India; 2 Department of Animal Nutrition, Institute of Animal Nutrition, Tamil Nadu Veterinary and Animal Sciences University, Chennai, Tamil Nadu, India; 3 Department of Animal Nutrition, Madras Veterinary College, Tamil Nadu Veterinary and Animal Sciences University, Chennai, Tamil Nadu, India

**Keywords:** hatchability, hatch weight, *in ovo* feeding, nanoparticles, and post hatch performance

## Abstract

**Background and Aim:**

Nanoparticles can bypass conventional physiological ways of nutrient distribution and transport across tissue and cell membranes, as well as protect compounds against destruction prior to reaching their targets. *In ovo* administration of nanoparticles, may be seen as a new method of nano-nutrition, providing embryos with an additional quantity of nutrients. The aim of the study is to examine the effect of *in ovo* supplementation of nano forms of zinc, copper and selenium on the hatchability and post hatch performance of broiler chicken.

**Materials and Methods:**

Nano form of zinc at 20, 40, 60 and 80 µg/egg, nano form of copper at 4, 8, 12 and 16 µg/egg and nano form of selenium at 0.075, 0.15, 0.225 and 0.3 µg/egg were *in ovo* supplemented (18^th^ day incubation, amniotic route) in fertile broiler eggs. Control group *in ovo* fed with normal saline alone was also maintained. Each treatment had thirty replicates. Parameters such as hatchability, hatch weight and post hatch performance were studied.

**Results:**

*In ovo* feeding of nano minerals were not harmful to the developing embryo and did not influence the hatchability. Significantly (p<0.05) best feed efficiency for nano forms of zinc (2.16), copper (2.46) and selenium (2.51) were observed, when 40, 4 and 0.225 µg/egg respectively were *in ovo* supplemented. Except in nano form of copper at 12 µg per egg which had significantly (p<0.05) highest breast muscle percentage there was no distinct trend to indicate that dressing percentage or breast muscle yield was influenced in other treatments.

**Conclusion:**

Nano forms of zinc, copper and selenium can be prepared at laboratory conditions. *In ovo* feeding of nano forms of zinc, copper and selenium at 18^th^ day of incubation through amniotic route does not harm the developing embryo, does not affect hatchability.

## Introduction

Indian poultry sector has been growing at around 8-10% annually over the last decade with broiler meat volumes growing at more than 10%, and table egg growing at 5-6% [1]. The growth of the industry can be attributed to the production performance of commercial poultry which has grown linearly every year, and the trend is likely to continue in future with the advent of development in the field of genetics, nutrition, biotechnology, developmental biology, etc. Early nutritional strategies offer the promise of sustaining progress in production efficiency and welfare of commercial poultry [2].

One-way to give hatchlings a nutritional jump start before they start eating feed is to feed them before they hatch (*In ovo* feeding). *In ovo* injection technology developed and patented by Uni and Ferket [3] provides a method to safely introduce external nutrients into developing embryos. During late embryogenesis, solutions injected into the amniotic fluid are subsequently swallowed, digested, and absorbed by the embryo before piping [4]. Rapid growth coupled with a high nutrient requirement, especially during late embryogenesis, may make *in ovo* feeding of supplemental nutrients beneficial to poultry. Supplementing the amnion with appropriate nutrients is a novel way to feed critical dietary nutrients to embryos. Mineral reserves in yolk decrease significantly from the day of setting; this leaves the embryo with low mineral reserves for the last period of incubation and probably leads to a mineral deficiency status of the embryo [5]. *In ovo* feeding of minerals has also gained importance as the high-metabolism, fast-growing broiler embryos may reach levels of mineral deficiency that can lead to metabolic disorders [6].

*In ovo* feeding could lead to improved digestive capacity, increased growth rate and feed efficiency, reduced post-hatch mortality and morbidity, improved immune response to enteric antigens, reduced incidence of developmental skeletal disorders, and increased muscle development and breast meat yield [7].

The *in ovo* injection of L-carnitine has shown many beneficial effects in post-hatch performance [8,9]. The protection level against *Salmonella enteritidis* was evaluated in chickens after *in ovo* treatment with different species of *Lactobacillus* spp. inoculated into the air cell or by immersion in broth culture [10]. *In ovo* fed birds exhibited higher glycogen reserves, body weight, pectoral muscle weight and body weight gain than control birds [11]. The *in ovo* administration of manan oligosaccharides (MOS) showed a short-term effect resulting in a hatching chick with more mature enterocytes in the small intestine and enhanced digestive capacity and epithelial barrier, which can, in turn, improve development and growth in the 1^st^ days after hatch. The beneficial effects of MOS began 72 h after *in ovo* administration and lasted at least until date of hatch [12].

Zinc plays a role in the development of the immune system of the broiler embryo [13,14]. In a large number of studies, additional zinc used in the diet of broilers has improved antibody production [15]. Zinc is crucial for normal development and function of cells mediating non-specific immunity such as neutrophils and natural killer cells [16]. Effects of dietary copper-loaded chitosan nanoparticle (CNP-Cu) supplementation on growth performance, hematological and immunological characteristics and the cecal microbiota in broilers were investigated. Results indicated that supplemental CNP-Cu could improve growth performance; affect the immune system [17]. Selenium supplementation in experimental animals has been shown to be associated with increases in natural killer cell activity, T-cell proliferation, lymphokine-activated killer cell activity, delayed-type hypersensitivity skin responses, and vaccine-induced immunity [18]. Examination of the effect of *in ovo* enrichment of phosphorus (P), calcium (Ca), iron (Fe), zinc (Zn), copper (Cu), and manganese (Mn) along with vitamins, amino acids and carbohydrates showed that the enrichment resulted in increased iron, zinc, copper, and manganese levels in the yolk even though the minerals are supplemented in the amniotic fluid [19]. This research study is the first of its kind in exploring the significant beneficial effects of nano forms of zinc, copper, and selenium when fed *in ovo*.

Nanoparticles have different physical and chemical characteristics compared to their larger equivalents because of a very high surface to volume ratio, physical activity, and chemical stability. The small size of nanoparticles allows for penetration inside tissues and even enables them to cross cell membranes. Nanoparticles can bypass conventional physiological ways of nutrient distribution and transport across tissue and cell membranes, as well as protect compounds against destruction before reaching their targets. *In ovo* administration of nanoparticles, acting as bioactive agents and as carriers of nutrients may be seen as a new method of nano-nutrition, providing embryos with bioactive compounds and/or with an additional quantity of nutrients or energy. Nutrient supplementation through *in ovo* was reported to be a more efficient when a compound was attached to nanoparticles (silver or gold), which delivered it inside the body tissues and cells [20]. It is with this background a research was carried out wherein the post-hatch performance of broiler chicken *in ovo* supplemented with nano forms of zinc, copper, and selenium was studied. Thus, the aim of this research study is to examine the effect of *in ovo* supplementation of nano forms of zinc, copper and selenium on the hatchability and post-hatch performance of broiler chicken.

## Materials and Methods

### Ethical approval

No Ethical Committee approval was necessary for this study as we conducted experiment on broiler chicken with a lifespan of about 5-6 weeks. However, we conducted experiment under very fine confinement without giving any undue stress to the birds.

### Experimental design

In an earlier study, Bakyaraj *et al*. [21] recommended *in ovo* feeding levels for zinc, copper and selenium as 80, 16 and 0.3 µg, respectively/egg. For this study, these levels were considered as 100% of the requirement of the respective minerals. The efficacy of the respective nano minerals *viz*. zinc, copper and selenium at four graded levels (25%, 50%, 75% and 100%) were tested. A control group wherein only normal saline was *in ovo* fed was maintained. Each egg was weighed and randomly distributed into respective treatment groups (Table-1) maintaining similar average weight across treatment groups. In each treatment, 30 eggs were set in the incubator in three separate groups so that the hatch record could be maintained for three replicates per treatment. Thus, each nano mineral had five treatments (four graded levels and one control).

**Table-1:** Treatment groups experimented to assess optimum level of nano forms of zinc, copper and selenium required to be fed *in ovo* to fertile broiler eggs.

Treatment	Normal saline ml/egg	Percent inclusion of nano form of minerals	Nano form of minerals µg/egg
Control	0.5 ml	0	0
Nano form of zinc	0.5 ml	25	20
	0.5 ml	50	40
	0.5 ml	75	60
	0.5 ml	100	80
Control	0.5 ml	0	0
Nano form of copper	0.5 ml	25	4
	0.5 ml	50	8
	0.5 ml	75	12
	0.5 ml	100	16
Control	0.5 ml	0	0
Nano form of selenium	0.5 ml	25	0.075
	0.5 ml	50	0.15
	0.5 ml	75	0.225
	0.5 ml	100	0.3

### Production of nano forms of zinc, copper, and selenium and their characterization

Nano form of zinc, copper, and selenium was produced in triplicate adopting the procedure as explained in this section. Nano form of zinc was produced by a chemical method using starch as a stabilizing agent. Starch solution (0.5%) was prepared and 20-50 ml of 0.2 M zinc acetate dihydrate was added with few drops of 0.2 M of sodium hydroxide. The pH was adjusted to 8.5 using 0.2 M NaOH. The contents were stirred continuously at 100°C. A milky white colloid was obtained; the colloid was stirred for 2 h and centrifuged at 9000 rpm for 15 min. The sediment was filtered and washed using initially acetone, followed by ethanol and water. After which the sediment was dried in hot air oven at 80°C for 3 h. Thus, produced nano form of zinc’s yield was determined and characterized [22].

Nano form of copper was produced by electrochemical method. An indigenous laboratory electrolysis unit was fabricated and feed grade copper sulfate solution was subjected to electrolysis using copper rods as anode and cathode. The flow of a steady current into the electrolytic cell caused the ionization and disassociation of copper sulfate solution which removed the copper from the anode and deposited it in the cathode. Such deposited copper, was collected dried, yield determined, and characterized [23].

Nano form of selenium was prepared by adopting the procedure of Razi *et al*. [24]. Selenium powder 0.1 g was mixed with 2.4 g sodium hydroxide in 40 ml of distilled water. The contents were maintained at a temperature of 140°C for 1 h. The contents were then cooled to room temperature, filtered and washed using water and ethanol. The residue was dried yield determined and characterized.

The size and zeta potential of the nano zinc, copper, and selenium produced was determined using particle size analyzer (Malvern make Model No. 2000). The zinc, copper and selenium content of the respective samples from each of the method were determined using Atomic Absorption Spectrophotometer (Perkin-Elmer, Model 3110, 1994) as per the procedure outlined in the reference manual.

### Fumigation and incubation

A total of 390 fertile broiler eggs (Vencobb 400) were procured, fumigation of all the eggs was carried out in a fumigation hood. Fumigation was done using 57 g of potassium permanganate (×1 concentration) and 85 ml of formalin (×1.5 concentration). The eggs were set in an incubator with setter temperature of 100°F and relative humidity of 85%. Eggs were candled on 7^th^ and 14^th^ day to remove infertile eggs.

### *In ovo* feeding procedure

On 18^th^ day of incubation, candling of eggs were carried out and amniotic route was marked and a small pinpoint hole was made in the broad end of the egg to remove the egg shell by using Topaz Engraver as egg driller and *in ovo* supplementation was done according to the treatments through the amniotic route using a 24G hypodermic needle (25 mm long) and the pinpoint hole was sealed using wax [25]. The eggs were placed back in incubator with hatcher temperature of 100°F and relative humidity of 90%.

### Parameters studied

Parameters studied included hatchability, hatch weight of chicks, chick weight is to egg weight ratio, post-hatch performance relating to weight gain, feed efficiency, and slaughter studies. The percent hatchability was determined using the following formulae:


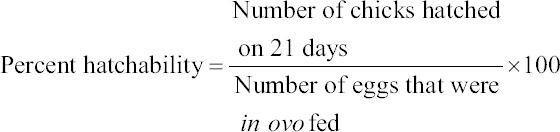


The hatch weight of chicks was determined by weighing the chicks in an electronic weighing balance and expressed in grams. The chick weight is to egg weight ratio was determined using the following formula:


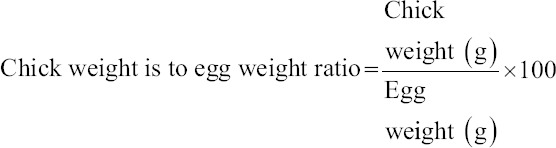


To study the post-hatch performance 20 chicks from each treatment were weighed, wings banded, maintained in their same respective groups and were reared for a period of 5 weeks. The experimental birds were housed in five-tiered, well-ventilated battery cages provided with artificial lighting.

The standard managemental practices were adopted, and they were uniform for all the treatment groups. All the chicks in the various treatments were fed *ad libitum* quantity of a common experimental ration. The chicks were fed with broiler starter ration from 0 to 3 weeks and broiler finisher ration from 4 to 5 weeks. Clean drinking water was provided *ad libitum*. The ingredient and nutrient composition of the experimental ration are presented in Table-2.

**Table-2:** Ingredient and nutrient composition of the experimental rations.

Ingredients	Broiler starter	Broiler finisher
Ingredient composition (%)		
Maize	50	45
Bajra	-	18
Soyabean meal	32	23
De-oiled rice bran	1	-
Fish meal	10	8
Oil	4.5	4
Mineral mixture	2	1.6
L-lysine	0.1	0.05
DL-methionine	0.15	0.10
Salt	0.25	0.25
Total	100.00	100.00
Nutrient composition		
Crude protein (%)	21.73	19.78
Metabolizable energy (kcal/kg)	3160	3252
Crude fiber (%)	3.00	2.85
Calcium (%)	1.31	1.22
Availablephosphorus (%)	0.49	0.46
Lysine (%)	1.21	0.94
Methionine (%)	0.50	0.31

Additives added per 100 kg feed - Vitamin AB_2_D_3_K - 0.01 g, Ultracil - 0.05 g, Unicox - 0.02 g, Tefroli - 0.05 g, Ultra B_12_-0.01 g, Perivac - 0.02 g, Spectra - 0.01 g, Larvadex - 0.05 g

Every day the left over feed and wastage that spilled outside the feed trough was collected and weighed so as to record the accurate feed intake in grams. The chicks were sex corrected to calculate weight gain and feed efficiency. The birds were weighed individually every week in a calibrated balance to document their weight gain which was expressed in grams. The feed efficiency per kilogram weight gain was calculated using the following formulae:


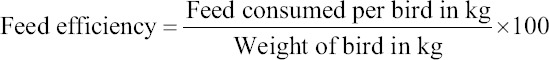


At the end of the trial (5 weeks of age), six birds from each treatment were selected randomly and slaughtered by decapitation to record the live weight, carcass weight, and giblets weight. The dressing percentage and giblet percentage were calculated. Breast muscle was separated, and breast muscle yield was recorded and expressed in terms of percent dressed weight.

### Statistical analysis

The design of all the experiments in this study was completely randomized design. Data were analyzed with analysis of variance as per procedure of statistical analysis system (SAS/SPPSS, 1999, version 10.0 for windows). When a significant difference (p<0.05) were detected, the multiple range test was used to separate the mean value.

## Results and Discussion

The product yield, particle size, zeta potential, mineral content in nano forms of zinc, copper, and selenium are presented in Table-3.

**Table-3:** Product yield, particle size, zeta potential and mineral content in nano forms of zinc, copper and selenium, respectively.

Parameters	Nano form minerals		

	Zinc	Copper	Selenium
Mean product yield (g/h)	1.0	1.0	0.1
Size (assessed through particle size analyser) nm*	78.3±0.35	72.3±0.27	74.9±0.28
Zeta potential (mV)*	−24.7±0.45	−27.2±0.27	−26.1±0.28
Mineral content (ppm)*	92.06±0.12	88.10±0.15	94.56±0.38

* Mean of three samples

The particle size of nano form of zinc, copper and selenium were below 100 nm confirming their nano size. Yadav *et al*. [26] reported a lower size (50 nm) for zinc oxide nanoparticles. Similar to this study Ramyadevi *et al*. [27] also reported 35-80 nm sized copper nano particles produced by polyol process. Zhang *et al*. [28] also had reported the size of nano red elemental selenium (Nano-Se) in the range from 20 to 60 nm.

The stability of the nano form of zinc, copper and selenium ascertained by their zeta potential, lies well within the stable limits *viz*. > +25 mV or < −25 mV [29]. The respective mineral content in nano forms of zinc, copper, and selenium were indicative of a high level of purity. The yield of the products produced was low. Earlier studies in the laboratory also evinced that production of nano forms of minerals by wet chemical method or electrochemical method was 160-200 times lower than that produced by physical method using ball mill [30]. However, the quantity produced through wet chemical (zinc and selenium), or electrochemical (copper) methods was sufficient to meet the *in ovo* feeding, and hence these methods were adopted. Nanoparticles can bypass conventional physiological ways of nutrient distribution and transport across tissue and cell membranes, as well as protect compounds against destruction prior to reaching their targets. In which case *in ovo* administration of nano particles, acting as bioactive agents and as carries of nutrients may be seen as a new method of nano-nutrition [20].

The effect of *in ovo* feeding of broiler eggs with nano form of zinc, copper and selenium at graded levels on egg weight, hatch weight of chicks and their ratio, hatchability percent is presented in Table-4.

**Table-4:** Effect of *in ovo* feeding of broiler eggs with nano form of zinc, copper and selenium at graded levels on egg weight, hatch weight of chicks and their ratio and hatchability percent*.

Treatments	Level of inclusion (%)	Level of inclusion (µg/egg)	Egg weight (g)^NS^	Hatch weight of Chicks (g)^NS^	Ratio of chick weight to egg weight^NS^	Hatchability percent^NS^
Control	0	0	60.73±0.68	47.52±0.72	78.24±0.37	96.66±3.33
Nano form zinc	25	20	60.31±1.05	47.30±0.86	78.81±1.84	96.29±3.70
	50	40	61.90±1.99	46.56±0.93	75.54±1.69	96.29±3.70
	75	60	60.93±0.88	46.29±0.68	76.01±0.62	92.96±3.53
	100	80	61.50±0.79	47.62±0.69	77.40±0.36	88.42±0.46
Nano form of copper	25	4	61.14±0.94	47.53±0.73	77.79±0.62	92.96±3.53
	50	8	60.76±0.68	47.17±0.68	77.62±0.58	92.96±3.53
	75	12	62.41±0.82	47.46±0.64	76.07±0.50	92.12±3.95
	100	16	59.82±0.95	46.14±0.77	77.39±1.55	92.96±3.53
Nano form of selenium	25	0.075	61.44±0.99	46.96±0.72	76.47±0.47	83.33±11.02
	50	0.15	62.10±0.90	47.04±0.81	76.03±1.72	83.33±11.02
	75	0.225	60.69±1.06	48.02±0.76	79.41±1.55	92.59±3.70
	100	0.3	60.78±0.73	47.69±0.63	78.56±1.00	88.88±6.41

* Mean of 25 observations. NS=Non-significant difference between treatments

No significant variation (p>0.05) existed in the egg weight, hatch weight of the chicks or their ratio and hatchability percent between the treatment groups (control and graded levels of nano form of zinc/copper/selenium) studied.

The hatchability percentage obtained in this study for nano forms of zinc, copper and selenium at graded levels were higher than that previous study reported by Bakyaraj *et al*. [21], who reported hatchability of 81.3% on *in ovo* feeding of selenium 0.3 µg, zinc 80 µg, copper 16 µg and manganese 120 mg/egg. They also reported a hatchability of 61.3% on *in ovo* feeding of selenium 0.3 µg, zinc 80 µg, iron 160 µg and iodine 0.7 µg/egg. The chick weights on hatch in this study for nano forms of zinc, copper and selenium at graded levels were similar to that reported by Bakyaraj *et al*. [21]. However, the chick weight to egg weight ratio was higher in the present study. More than minerals, *in ovo* feeding of energy sources or protein sources is likely to improve hatch weight of chicks as they would supplement the crucial needs of these nutrients during the vital period of hatching [31]. The degree of response to *in ovo* feeding may depend on genetics, breeder hen age, egg size, and incubation conditions [32]. Salmanzadeh [33] reported a reduced hatchability on *in ovo* injection of glucose and attributed it to the development of allergic reactions under air sac that stopped the respiration of the embryo causing its death. Whether such type of change occurred with regard to *in ovo* feeding of coarse form of minerals when particle size was larger is uncertain. However, nano form of minerals owing to its particle size, have an ability to remain in colloidal state and might have not caused harm to the embryo.

The effect of *in ovo* feeding of broiler eggs with nano form of zinc, copper and selenium at graded levels on overall weight gain, feed efficiency and mortality of broilers at 0-5 weeks is presented in Table-5.

**Table-5:** Effect of *in ovo* feeding of broiler eggs with nano form of zinc, copper and selenium at graded levels on overall weight gain, feed efficiency and mortality of broilers (0-5 weeks)*.

Treatments	Level of inclusion	Level of inclusion (µg/egg)	Chick weight (g)^NS^	Final weight (g)	Weight gain (g)	Feed efficiency	Mortality percent^NS^
Control	0	0	47.52±0.72	1374.0^a^±22.82	1326.63^a^±22.63	2.30^b^±0.04	0
Nano form of zinc	25	20	47.28±0.78	1360.93^a^±26.15	1313.26^a^±25.84	2.33^b^±0.04	4.5
	50	40	47.01±0.90	1419.29^b^±17.28	1372.05^b^±17.64	2.16^a^±0.03	4.5
	75	60	46.43±0.69	1300.92^a^±8.69	1254.55^a^±8.86	2.42^bc^±0.01	0
	100	80	47.44±0.65	1348.62^a^±23.01	1301.09^a^±22.93	2.89^c^±0.06	0
Control	0	0	47.52±0.72	1374.0^b^±22.82	1326.63^b^±22.63	2.30^a^±0.04	0
Nano form of copper	25	4	47.68±0.67	1315.91^a^±14.23	1268.12^a^±14.01	2.46^a^±0.02	0
	50	8	47.07±0.64	1388.94^b^±22.53	1341.76^b^±22.34	2.55^a^±0.04	0
	75	12	47.17±0.63	1377.94^b^±21.46	1330.47^b^±21.49	2.68^b^±0.04	0
	100	16	46.08±0.70	1336.67^ab^±14.75	1289.88^ab^±14.96	2.46^a^±0.03	4.5
Control	0	0	47.52±0.72	1374.0^a^±22.82	1326.63^a^±22.63	2.30^a^±0.04	0
Nano form of selenium	25	0.075	46.56±0.72	1453.97^b^±22.54	1406.86^b^±22.38	2.90^c^±0.04	0
	50	0.15	46.92±0.74	1470.90^b^±15.59	1423.56^b^±15.82	2.85^c^±0.02	0
	75	0.225	47.85±0.70	1355.57^a^±16.96	1307.55^a^±16.92	2.51^a^±0.03	4.5
	100	0.3	47.94±0.72	1386.10^a^±19.49	1338.41^a^±19.44	2.66^b^±0.03	4.5

* Mean of 25 observations. Means bearing different superscripts within columns differ significantly (p<0.05). NS=Non significant difference between treatments

No significant variation (p>0.05) existed in the chick weight or mortality percentage between control and graded levels of nano form of zinc studied. Significantly (p<0.05) highest final weight and weight gain was observed in 50% inclusion of nano form of zinc. This inclusion level also resulted in significantly (p<0.05) the best feed efficiency.

No significant variation (p>0.05) existed in the chick weight or mortality percentage between control and graded levels of nano form of copper studied. Significantly (p<0.05) lowest final weight and weight gain was observed in 25% (4 µg/egg) and 100% (16 µg/egg). The feed efficiency was comparable between control, 25% (4 µg/egg), 50% (8 µg/egg), and 100% (16 µg/egg) inclusion level of nano form of copper. The inclusion level of nano form of copper at 75% (12 µg/egg) revealed a significantly (p<0.05) highest feed efficiency value. Among the various graded level of inclusion of nano form of copper, 25% (4 µg/egg) inclusion proved to be the best in terms of feed efficiency.

No significant variation (p>0.05) existed in the chick weight or mortality percentage between control and graded levels of nano form of selenium studied. The final weight and weight gain were significantly (p<0.05) highest in 25% (0.075 µg/egg) and 50% (0.15 µg/egg) inclusion level of nano form of selenium. However, the feed efficiency value was significantly (p<0.05) lower, and therefore, the best in control and 75% (0.225 µg/egg) inclusion level of nano form of selenium. Nano form of selenium included at 75% (0.225 µg/egg) level lead to a significantly (p<0.05) lowest feed efficiency. Among the various graded level of inclusion of nano form of selenium, 75% inclusion (0.225 µg/egg) proved to be the best in terms of feed efficiency.

Similar to the results obtained in this study wherein the feed efficiency on *in ovo* feeding of nano form of copper and selenium showed no improvement over that of control, Bakyaraj *et al*. [21] also reported that there was no significant (p<0.05) difference observed in feed conversion ratio of *in ovo* trace elements injected chicks.

The effect of *in ovo* feeding of broiler eggs with nano form of zinc, copper and selenium at graded levels on dressing, giblet and breast muscle percentage of broilers at 0-5 weeks is presented in Table-6.

**Table-6:** Effect of *in ovo* feeding of broiler eggs with nano form of zinc, copper and selenium at graded levels on dressing, giblet and breast muscle percentage of broilers (0-5 weeks)*.

Treatments	Level of inclusion	Level of inclusion (µg/egg)	Dressing percentage	Giblet percentage	Breast muscle percentage^NS^
Control	0	0	66.26^bc^±0.82	4.84^a^±0.12	27.25±0.66
Nano form of zinc	25	20	59.84^a^±1.92	5.07^ab^±0.18	30.32±0.96
	50	40	66.33^bc^±1.38	5.66^c^±0.17	30.24±1.52
	75	60	62.44^ab^±1.23	5.16^ab^±0.15	30.33±2.46
	100	80	68.39^c^±1.85	5.45^bc^±0.17	26.72±0.93
Control	0	0	66.26±0.82	4.84±0.12	27.25^a^±0.66
Nano form of copper	25	4	65.28±2.09	5.51±0.09	26.74^a^±0.48
	50	8	62.84±0.96	4.98±0.39	26.73^a^±0.82
	75	12	63.94±0.95	5.17±0.07	29.31^b^±0.63
	100	16	65.45±0.74	5.14±0.17	26.12^a^±0.64
Control	0	0	66.26^b^±0.82	4.84^a^±0.12	27.25±0.60
Nano form of selenium	25	0.075	64.70^ab^±0.33	5.65^c^±0.25	27.34±0.3
	50	0.15	66.31^b^±0.77	5.43^bc^±0.16	27.11±0.28
	75	0.225	63.17^a^±0.51	5.06^ab^±0.05	27.06±0.28
	100	0.3	64.14^a^±0.53	5.10^ab^±0.16	26.30±0.39

* Mean of six observations. Means bearing different superscripts within columns differ significantly (p<0.05).

NS=Non-significant variation between treatments

The effect of *in ovo* feeding of broiler eggs with nano form of zinc revealed that dressing percentage was significantly (p<0.05) higher in 100% inclusion (80 µg/egg) level of nano form of zinc. However, significantly (p<0.05) highest giblet percentage was in 50% inclusion (40 µg/egg) level of nano form of zinc. No significant variation (p>0.05) existed in breast muscle percentage among the treatment groups (control and graded levels of nano form of zinc) studied.

The effect of *in ovo* feeding of broiler eggs with nano form of copper revealed that no significant variation (p>0.05) existed in dressing percentage and giblet percentage between control and graded levels of nano form of copper studied. However, significantly (p<0.05) higher breast muscle percentage was in 75% inclusion (12 µg/egg) level of nano form of copper.

The effect of *in ovo* feeding of broiler eggs with nano form of selenium at graded levels revealed significantly highest (p<0.05) dressing percentage in control and 50% inclusion (0.15 µg/egg) level of nano form of selenium. However, significantly highest (p<0.05) giblet percentage was observed in 25% inclusion level (0.075 µg/egg) of nano form of selenium. No significant variation (p>0.05) existed in breast muscle percentage between control and graded levels of nano form of selenium studied. Increasing protein above requirement enhanced the activation of components related to translation initiation in the neonate chick muscle but the role of trace elements is not clear [34].

## Conclusion

Stable forms of nano sized zinc and selenium with high purity can be produced by wet chemical method and stable nano sized copper with high purity can be produced by electrochemical method. *In ovo* feeding of nano forms of zinc, copper and selenium at the 18^th^ day of incubation through amniotic route does not harm the developing embryo and does not affect hatchability. Nano forms of zinc, copper and selenium gave best feed efficiency at certain inclusion levels. There existed no trend to indicate that the dressing percentage was influenced by different levels of nano forms of minerals. The breast muscle percentage was higher for nano form of copper at certain inclusion level. The research clearly shows that nano minerals are not harmful to the embryo and can be used to improve the post-hatch performance of broiler chicken. However, further many advanced researches are required to explore further beneficial effects and safety of nano forms of minerals.

## Authors’ Contributions

The present study was a part of original research work by PPJ during his M.V.Sc., thesis program under the eminent guidance of CV. CV and VB conceptualized the aim of the study, designed, planned and supervised the experiment. Collection of samples, execution of experimental study was done by PPJ. Analysis of data, interpretation of the results and drafting of the manuscript was done by PPJ, CV, and VB. All authors read and approved the final manuscript.
